# Association between ketone body levels and chronic liver disease: Epidemiological studies and potential mechanisms

**DOI:** 10.3934/publichealth.2026006

**Published:** 2026-01-07

**Authors:** Zeping Liu, Yihu Zheng, Yi Li

**Affiliations:** 1 West China School of Medicine, Sichuan University, Chengdu, China; 2 College of Computer Science, Sichuan University, Chengdu, China; 3 Department of General Surgery, the First Affiliated Hospital of Wenzhou Medical University, China; 4 Department of Laboratory Medicine, West China Hospital, Sichuan University, No. 37 Guoxue Xiang, Wuhou District, Chengdu, Sichuan, China

**Keywords:** chronic liver disease, ketone body, acetone, acetoacetates, β-hydroxybutyrate

## Abstract

Chronic liver disease encompasses conditions such as metabolic dysfunction-associated steatotic liver disease, metabolic dysfunction-associated steatohepatitis, alcohol-related liver disease, and chronic viral hepatitis, being a major cause of cirrhosis and hepatocellular carcinoma. Ketone bodies, crucial hepatic energy substrates, may serve as biomarkers in CLD progression. This review investigated the association between chronic liver diseases and ketone body levels. Studies indicate that ketone body levels may serve as biomarkers for chronic liver diseases. Patients with metabolic dysfunction-associated steatotic liver disease have elevated ketone levels. However, findings are inconsistent in obese populations and metabolic dysfunction-associated steatohepatitis. The relationship between ketone body levels and other chronic liver diseases, such as alcohol-related liver disease and chronic viral hepatitis, remains unclear, necessitating further research. The review also discussed potential mechanisms linking ketone metabolism abnormalities to chronic liver disease pathogenesis. Understanding the role of ketone bodies in chronic liver disease is essential for developing targeted interventions, and future research should employ large-scale databases and causal inference methods to clarify these associations.

## 1. Introduction

Chronic liver disease (CLD) is a significant global public health concern, characterized by diverse etiologies, including metabolic dysfunction-associated steatotic liver disease (MASLD, formerly known as non-alcoholic fatty liver disease, NAFLD), metabolic dysfunction-associated steatohepatitis (MASH, formerly known as non-alcoholic steatohepatitis, NASH), alcohol-related liver disease (ALD), chronic viral hepatitis, and hepatic fibrosis. These conditions can progress to cirrhosis and hepatocellular carcinoma (HCC), profoundly impacting patients' quality of life and survival rates. Therefore, gaining a comprehensive understanding of the pathophysiological mechanisms underlying CLD and identifying effective biomarkers and therapeutic strategies constitute pivotal directions in current research.

Ketone bodies (e.g., β-hydroxybutyrate and acetoacetate) are crucial energy substrates that are synthesized through ketogenesis in hepatic cells, particularly in conditions such as diabetes, starvation, or low-carbohydrate diets. Emerging research suggests that the dysregulation of ketone body metabolism is not merely a consequence but may be integrally involved in the pathogenesis of various CLDs. For instance, altered ketone body levels have been correlated with different stages and types of CLD [1],[2], and elevated levels have been associated with increased mortality in MASLD patients [3], hinting at their potential diagnostic and prognostic value.

This review systematically evaluates the epidemiological evidence linking ketone body levels to major CLDs and explores the potential mechanistic pathways underlying this relationship. By integrating findings across MASLD/MASH, ALD, viral hepatitis, and HCC, we aim to clarify the role of ketone bodies in CLD and highlight directions for future research.

## 2. Biological basis of ketone body metabolism

**Figure 1.**
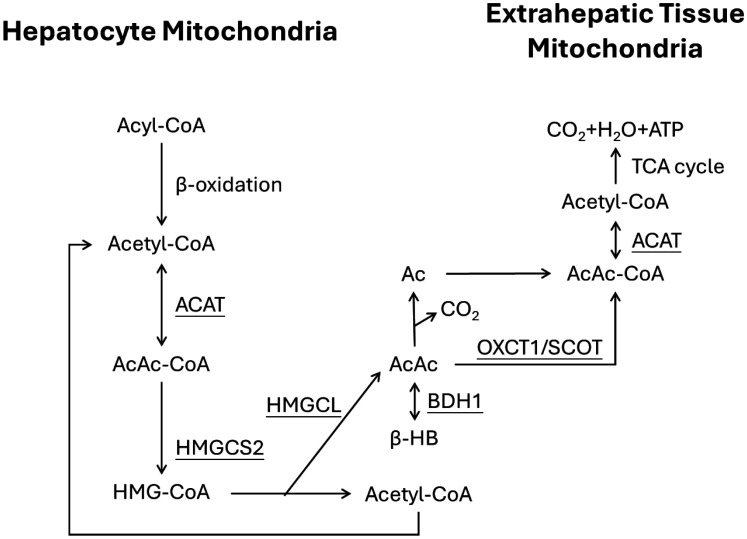
Pathways of ketogenesis in the liver and ketolysis in extrahepatic tissues. [Ac, acetone; AcAc, acetoacetate; AcAc-CoA, acetoacetyl CoA; β-HB, β-hydroxybutyrate; HMG-CoA, hydroxymethylglutaryl-CoA; TCA cycle, tricarboxylic acid cycle. Underlined are key enzymes: ACAT, acetyl-CoA acetyltransferase; BDH1, β-hydroxybutyrate dehydrogenase 1; HMGCL, 3-hydroxy-3-methylglutaryl-CoA lyase; HMGCS2, 3-hydroxy-3-methylglutaryl-CoA synthase 2; OXCT1, 3-Oxoacid CoA-Transferase 1 (also known as SCOT, succinyl-CoA:3-ketoacid CoA transferase)].

Ketone bodies are specific intermediate metabolites produced during the hepatic β-oxidation of fatty acids, comprising three compounds: acetone, acetoacetate, and β-hydroxybutyrate. Ketone body metabolism primarily involves ketogenesis and ketolysis. Ketogenesis is the process by which fatty acids are converted into acetoacetate and β-hydroxybutyrate within the mitochondria of hepatocytes in the periportal region (Figure 1). Once generated through ketogenesis in the liver, ketone bodies can exit hepatocytes and enter the bloodstream. Circulating through the blood, they reach extrahepatic tissues where, within the mitochondria of these extracellular organs, they undergo ketolysis, oxidizing to produce energy. This energy is utilized to fuel metabolic activities within various organs and cells.

Ketone bodies are produced in large amounts by the liver during starvation, fasting, or conditions like diabetes. They are then transported to organs and tissues throughout the body. These include the brain, heart, kidneys, and skeletal muscles. Ketone bodies serve as an alternative energy source to maintain the energy supply needs of the body's tissues and cells in lieu of glucose. When an excess of ketone bodies is produced, the presence of both acetoacetate and β-hydroxybutyrate, both of which are moderately strong acids, can lead to the body being in an acidic environment. This results in the onset of ketosis, and in severe cases, may lead to ketoacidosis [4]. Additionally, the three constituent components of ketone bodies can also serve as metabolic signals to control various cellular metabolic processes. For example, β-hydroxybutyrate can act as a signaling molecule through binding to G-protein-coupled receptors and regulating potassium ion channels [5]–[8].

## 3. Mechanistic insights into ketone body dysregulation in chronic liver disease

The reasons for the correlation between ketone body levels and CLD are currently unclear [9]. The relationship between ketone body levels and MASLD has been studied extensively. One mainstream hypothesis suggests that this correlation mainly originates from the liver's robust compensatory function [10]. The liver, as an organ with strong compensatory capacity, can handle three times the normal amount of fat metabolism through ketogenesis [11],[12]. Therefore, before detectable pathological changes occur, the liver's ketogenesis enters a compensatory phase, enhancing the metabolism of excess fatty acids [13]. In other words, compared to healthy individuals, patients with MASLD promote compensatory ketogenesis to break down the accumulated fatty acids, resulting in elevated levels of ketone bodies [3]. This conclusion has been validated in a mouse model, where both short-term high-fat diet-induced MASLD mice and fasted mice showed elevated blood ketone body levels compared to normal mice [14],[15]. Several studies indicate that although ketone body levels in individuals with MASLD may be elevated compared to the normal population [3], obese patients with MASLD exhibit a downward trend in ketone body levels compared to obese individuals without MASLD [16]. This may be due to the association between blood ketone body levels and insulin sensitivity [17],[18]. Mouse studies indicate that initial hepatic ketogenesis is enhanced in mice fed a high-fat diet [14], while prolonged high-fat feeding gradually reduces ketogenesis [19].

As the disease progresses to MASH, hepatic ketogenesis becomes dysfunctional. Prolonged excessive fatty acid metabolism leads to lipotoxicity from accumulated lipids, causing mitochondrial overload and dysfunction [20]. This impairs β-oxidation and ultimately disrupts hepatic ketogenesis, which may manifest as a decrease in ketone body levels in peripheral blood.

When patients are in a stable state of ALD, their blood β-hydroxybutyrate levels are lower [21]. Mouse experiments have shown that inhibiting the production of β-hydroxybutyrate exacerbates ethanol-induced liver damage, while intraperitoneal injection of β-hydroxybutyrate reverses it. This suggests that a decrease in β-hydroxybutyrate may be a cause of alcohol-related hepatitis [21]. This protective effect may be related to the Hcar2 receptor on liver macrophages. Hcar2 is a G protein–coupled receptor that can be activated by β-hydroxybutyrate. In addition, long-term alcohol consumption downregulates the expression of carnitine palmitoyltransferase-1A, a crucial rate-limiting enzyme in β-oxidation, impairing β-oxidation [22]. This may also explain the decrease in ketone body levels in chronic ALD. On the other hand, heavy drinkers are more prone to developing ketoacidosis. Dysfunction in ketogenesis among heavy drinkers may be induced by inadequate and unbalanced dietary intake, malnutrition, smoking, dehydration, and endocrine metabolic disturbances caused by ethanol interference [23]. Under these circumstances, the body is prone to hepatic gluconeogenesis inhibition, depletion of hepatic glycogen stores, hypoglycemia, and enhanced ketogenesis. Furthermore, alcohol can reduce hepatic gluconeogenesis and lead to decreased insulin secretion, enhanced lipolysis, impaired fatty acid flux to mitochondria, and compromised fatty acid oxidation. All of these effects further potentially promote ketogenesis. When individuals who chronically consume large amounts of alcohol suddenly drink on an empty stomach, their levels of growth hormone, adrenaline, cortisol, and glucagon increase. These hormonal changes also stimulate hepatic ketogenesis [24],[25].

When the body enters a state of decompensated cirrhosis or liver failure, it responds to extreme pathophysiological conditions with a stress response. Multiple factors may lead to inadequate food intake in patients, significant fluid loss, and the occurrence of starvation ketoacidosis.

For HCC, the possible mechanism for the elevated ketone body levels in HCC patients [26] may be that tumor cells require large amounts of energy for rapid growth, and the energy provided by ordinary glucose cannot meet the needs of tumor cells. Therefore, they rely on ketones as an alternative fuel to further support their energy requirements for growth [27],[28].

## 4. Epidemiological evidence of ketone body metabolism in CLD

### 4.1. Literature search strategy

This narrative review aimed at comprehensively synthesizing current evidence on ketone bodies in chronic liver disease. A systematic search of the PubMed databases was conducted for publications up to October 2024. The search combined keywords related to ketone body metabolism (“ketone bodies”, “β-hydroxybutyrate”, “acetoacetate”, “acetone”, and “ketogenesis”) and major chronic liver diseases (“metabolic dysfunction-associated steatotic liver disease”, “MASLD”, “non-alcoholic fatty liver disease”, “NAFLD”, “metabolic dysfunction-associated steatohepatitis”, “non-alcoholic steatohepatitis”, “alcohol-related liver disease”, “hepatitis”, “hepatic fibrosis”, “liver fibrosis”, “liver cirrhosis”, “hepatic cirrhosis”, “liver cancer”, and “hepatocellular carcinoma”).

**Figure 2.**
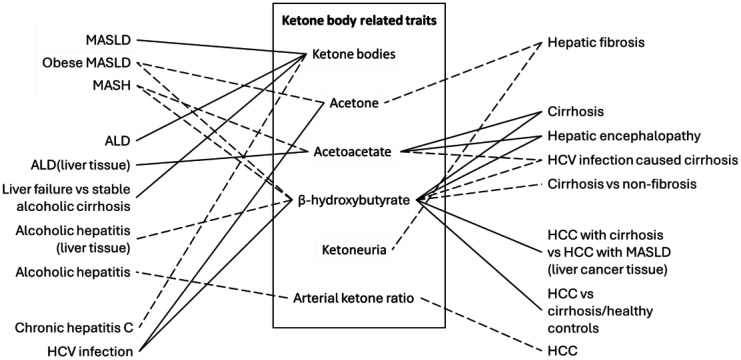
Summary of the associations between ketone bodies, related traits, and CLD based on epidemiological studies. MASLD, metabolic dysfunction-associated steatotic liver disease; MASH, metabolic dysfunction-associated steatohepatitis; HCV, hepatitis C virus; ALD, alcohol-related liver disease; HCC, hepatocellular carcinoma. The solid line indicates a positive association; the dashed line indicates a negative association.

The selection process prioritized original human observational studies (cross-sectional, cohort, and case-control) that reported quantitative data on circulating or hepatic ketone body levels. Reviews, mechanistic preclinical studies, and clinical trials were also consulted for context and theoretical frameworks. Conference abstracts without full data were generally excluded. To ensure comprehensive coverage, the reference lists of key articles were manually screened to identify additional pertinent publications (snowball sampling). A summary of the selected studies is shown in Table 1 and Figure 2.

**Table 1.** Overview of the epidemiological studies on ketone body levels and CLD.

CLD type	Reference	Study design and population	Tissue and platform	Fasting status	Adjustment variables	Findings
MASLD or MASH	Fotakis C et al., 2023 [29]	Case-control study. General population (*N* = 210): no steatosis (*n* = 39); mild steatosis (*n* = 119); moderate steatosis (*n* = 43); severe steatosis (*n* = 9).	Serum (^1^H-NMR)	Overnight fasting (12 h)	BMI	The decline in serum acetoacetate and acetone levels is associated with the risk of MASLD in females but not in males.
	Post A et al., 2021 [3]	Case-control study and cohort study. General population (*N* = 6297, middle-aged and elderly cohort): MASLD (elevated FLI, *n* = 1970); controls (*n* = 4327).	Plasma (^1^H-NMR)	Overnight fasting	Age, sex, lifestyle (alcohol, smoking), metabolic syndrome components, T2D, medication	MASLD patients exhibit elevated plasma ketone body levels compared with non-MASLD individuals, which correlates with an increased risk of all-cause mortality.
	Robinson EJ et al., 2021 [30]	Cross-sectional study. Obese women undergoing bariatric surgery (*N* = 71): hepatic steatosis (*n* = 39); non-MASLD controls (*n* = 32).	Serum (^1^H-NMR)	Not strictly controlled	Matched for BMI and underlying diseases	Serum β-hydroxybutyrate and acetone were significantly lower in obese women with steatosis compared to non-MASLD obese controls.
	Mey JT et al., 2020 [17]	Case-control study. Obese adults (*N* = 22); MASLD (*n* = 15); non-MASLD controls (*n* = 7).	Plasma (^1^H-MRS / Enzymatic)	Overnight fasting (12 h)	Correcting experimental variables in vitro and relying on pre-design balancing to reduce confounding	Plasma β-hydroxybutyrate was significantly reduced in obese MASLD patients compared to obese controls.
	Männistö VT et al., 2015 [31]	Cross-sectional study. Obese subjects (*N* = 116) classified by histology: normal liver (*n* = 32); simple steatosis (*n* = 19); MASH (*n* = 25); obese controls (*n* = 40).	Serum (^1^H-NMR)	Overnight fasting (12 h)	Type 2 Diabetes status	Serum acetoacetate and β-hydroxybutyrate were lower in MASH patients compared to those with simple steatosis.
	Croci I et al., 2013 [16]	Case control study. Mixed BMI cohort (*N* = 35): overweight/obese MASLD (*n* = 20); lean non-MASLD controls (*n* = 15).	Plasma (enzymatic assay)	Overnight fasting (10–12 h)	Age, sex, BMI, body fat, fat-free mass	Overweight/obese MASLD patients exhibited lower plasma β-hydroxybutyrate levels compared to lean non-MASLD controls.
	Kotronen A et al., 2009 [32]	Cross-sectional study. General population (*N* = 58): stable MASLD (*n* = 29); controls (*n* = 29).	Serum (enzymatic assay)	Overnight fasting	Age, gender, BMI	No statistically significant difference in serum β-hydroxybutyrate between stable MASLD patients and controls.
ALD	Chen Y et al., 2018 [21]	Case-control study. Surgical patients (*N* = 25): alcoholic hepatitis (*n* = 10); controls (hepatic metastases, *n* = 15).	Liver tissue (colorimetric assay)	N/A (surgical specimens)	None	Hepatic β-hydroxybutyrate concentration was reduced in the liver tissue of patients with alcoholic hepatitis.
	Cao D et al., 2017 [2]	Cross-sectional study. Cirrhotic patients with HCC (*N* = 60): ALD-HCC (*n* = 20); HBV-HCC (*n* = 20); HCV-HCC (*n* = 20).	Liver tissue (HRMAS ^1^H-NMR)	Overnight fasting (preoperative)	None (groups matched)	ALD-HCC patients exhibited significantly higher tissue acetoacetate levels compared to those with HBV- or HCV-related infection.
	YoKoyama A et al., 2014 [23]	Cross-sectional study. Alcoholic men in detoxification center (*N* = 1588) (active drinkers admitted for treatment).	Urine and serum (dipstick/enzymatic)	Upon admission (acute state)	Age, BMI, alcohol intake, time since last drink	Active heavy drinking and acute withdrawal states in ALD are associated with elevated blood ketone body levels.
	Amathieu R et al., 2014 [33]	Cross-sectional study. Cirrhosis patients (*N* = 123): stable cirrhosis (*n* = 93); acute-on-chronic liver failure (ACLF, *n* = 30).	Serum (^1^H-NMR)	Fasting (stable) *vs*. admission (ACLF)	None (multivariate OPLS-DA)	Serum ketone levels were significantly higher in ACLF patients compared with those with stable cirrhosis.
	Saibara T et al., 1994 [34]	Cohort study. Alcoholic hepatitis (*N* = 63): included 15 with liver cirrhosis (severe acute presentation).	Plasma (enzymatic/Ketorex)	Non-fasting (glucose/TPN-loaded)	none	Patients have a higher likelihood of survival if their AKBR rises above 0.7 within 72 h of hospitalization. The AKBR may serve as a potential marker for fatal complications and adverse prognosis in patients with alcoholic hepatitis.
	Stewart A et al., 1983 [35]	Case-control study. Stable ALD (*N* = 24): alcoholic cirrhosis (*n* = 10); alcoholic hepatitis (*n* = 5); controls (*n* = 9).	Whole blood (enzymatic assay)	Overnight fasting	None	Ketone body concentrations are similar in stable ALD patients and controls.
Chronic Viral Hepatitis	Gaia M et al., 2019 [36]	Cross-sectional study. Chronic viral hepatitis (*N* = 160): HCV infection (*n* = 67); HBV infection (*n* = 50); healthy controls (*n* = 43).	Serum (^1^H-NMR)	Fasting	None (multivariate analysis)	Serum β-hydroxybutyrate was increased in HCV patients compared to HBV patients and healthy controls.
	Cao D et al., 2017 [2]	cross-sectional study. Cirrhotic patients with HCC (*N* = 60): ALD-HCC (*n* = 20); HBV-HCC (*n* = 20); HCV-HCC (*n* = 20).	Liver tissue (^1^H-NMR)	Overnight fasting (preoperative)	None (groups matched)	Compared to patients with HBV-related primary HCC and those with ALD-associated primary HCC, individuals with HCV-related primary HCC exhibit significantly reduced tissue acetoacetate levels.
	Embade N et al., 2016 [37]	Cross-sectional study. Chronic hepatitis C (*N* = 57): non-fibrotic (F0, *n* = 30); compensated cirrhotic (F4, *n* = 27).	Serum (^1^H-NMR)	Overnight fasting	None (multivariate analysis)	Serum levels of acetoacetate and β-hydroxybutyrate are significantly reduced in patients with cirrhosis.
	Sato C et al., 2013 [38]	Cohort study. Chronic Hepatitis C (*N* = 38): chronic hepatitis C patients (*n* = 30); healthy volunteers (*n* = 8).	Serum (enzymatic assay)	Overnight (12 h) and prolonged (15 h)	Stratified by viral load and HOMA-IR	Patients with chronic hepatitis C exhibit a significant decrease in total ketone body levels in serum.
Hepatic Fibrosis	Lim K et al., 2021 [39]	Cross-sectional study. MASLD cohort (*N* = 6202) (excluded prediabetes and diabetes).	Urine (Dipstick semi-quantitative)	Overnight fasting (8 h)	Sex, obesity, hypertension, lipid profile, hsCRP, HOMA-IR	Ketoneuria is negatively correlated with the degree of liver fibrosis and is unrelated to traditional metabolic factors.
	Robinson EJ et al., 2021 [30]	Cross-sectional study. Obese women undergoing bariatric surgery (*N* = 71): hepatic steatosis (*n* = 39); non-MASLD controls (*n* = 32).	Serum (^1^H-NMR)	Not strictly controlled	Matched for BMI and underlying diseases	Serum levels of β-hydroxybutyrate and acetone are significantly lower in liver fibrotic patients compared to those in non-MASLD individuals, while they do not differ significantly from levels observed in individuals with simple MASLD.
Cirrhosis	Dabos KJ et al., 2015 [1]	Cross-sectional study. Study participants (*N* = 53): stable compensated cirrhosis (*n* = 18); cirrhosis with hepatic encephalopathy (*n* = 18); healthy controls (*n* = 17).	Plasma (^1^H-NMR)	Post-prandial (2–3 h after meal)	None (matched for age/sex)	Both acetoacetate and β-hydroxybutyrate were elevated in cirrhotics *vs*. controls. However, hepatic encephalopathy patients exhibited higher acetoacetate but lower β-hydroxybutyrate compared to stable cirrhosis.
	Liu Y et al., 2014 [26]	Cross-sectional study. Clinical cohort (*N* = 103): liver cirrhosis (*n* = 42); HCC (*n* = 43); healthy volunteers (*n* = 18).	Serum (^1^H-NMR and LC-MS)	Not explicitly reported	None (Random Forest classification)	Significant differences in serum ketone profiles distinguished cirrhosis from healthy controls and HCC.
	Amathieu R et al., 2014 [33]	Cross-sectional study. Cirrhosis patients (*N* = 123): stable cirrhosis (*n* = 93); ACLF (*n* = 30).	Serum (^1^H-NMR)	Fasting (stable) *vs*. admission (ACLF)	None (multivariate OPLS-DA)	In severe ALD patients, the serum levels of acetoacetic acid and β-hydroxybutyrate in liver failure patients are higher than those in stable cirrhosis patients.
HCC	Sasaki R et al., 2018 [40]	Clinical trial. HCC patients (*N* = 68): undergoing TACE treatment pre- *vs*. post-treatment comparison	Serum (enzymatic assay)	Fasting	Fib-4 index, tumor size	After TACE treatment, HCC patients show reduced blood ketone levels.
	Teilhet C et al., 2017 [41]	Case-control study. HCC patients (*N* = 28): HCC with cirrhosis (*n* = 9); HCC without cirrhosis (*n* = 19).	Serum (Enzymatic)	Fasting	None	HCC patients with cirrhosis have higher β-hydroxybutyrate levels in their liver cancer tissues than those with HCC and MASLD.
	Liu Y et al., 2014 [26]	Cross-sectional study. Clinical Cohort (*N* = 103): liver cirrhosis (*n* = 42); HCC (*n* = 43); healthy volunteers (*n* = 18).	Serum (^1^H-NMR and LC-MS)	Not explicitly reported	None (Random Forest classification)	HCC patients show significantly higher serum β-hydroxybutyrate levels compared to cirrhosis patients and healthy controls.
	Yan LN et al., 2007 [42]	Cohort study. Primary HCC (*N* = 2143) (surgical candidates undergoing hepatectomy).	Arterial plasma (colorimetric)	Glucose-loaded (non-fasting)	None (grouped by AKBR)	With proper management, mitochondrial function could gradually recover, leading to an increase in AKBR to above 0.7, and patients would recover gradually. It is believed that the arterial body ketone ratio can serve as an indicator reflecting the postoperative metabolic status of patients.

Note: ACLF, acute-on-chronic liver failure; AKBR, arterial ketone body ratio; ALD, alcohol-related liver disease; BMI, body mass index; CLD, chronic liver disease; FLI, fatty liver index; HBV, hepatitis B virus; HCC, hepatocellular carcinoma; HCV, hepatitis C virus; HOMA-IR, homeostatic model assessment for insulin resistance; HRMAS, high-resolution magic angle spinning; hsCRP, high-sensitivity C-reactive protein; LC-MS, liquid chromatography–mass spectrometry; MASLD, metabolic dysfunction-associated steatotic liver disease; MASH, metabolic dysfunction-associated steatohepatitis; MRS, magnetic resonance spectroscopy; NMR, nuclear magnetic resonance; OPLS-DA, orthogonal partial least squares discriminant analysis; T2D, type 2 diabetes; TACE, transcatheter arterial chemoembolization; TPN, total parenteral nutrition.

### 4.2. Ketone body levels with MASLD and MASH

MASLD is a clinical-pathological syndrome characterized primarily by hepatic steatosis and metabolic syndrome. MASLD is associated with an increased incidence of metabolic diseases, affecting approximately 25% of the population in developed countries, making it the fastest-growing liver disease worldwide [10],[43]. Without intervention, MASLD may progress to irreversible MASH, liver fibrosis, and even cirrhosis and HCC, imposing a substantial economic burden on the global economy [10],[43].

In recent years, the role of ketone body levels in MASLD has garnered widespread attention among researchers. Relevant studies can be broadly categorized into two types. One focuses on investigating the differences in ketone body levels between individuals with MASLD and those without MASLD in the general population, while the other delves into exploring the correlation between ketone body levels and MASLD specifically within the obese population. For instance, Post et al. conducted a large-scale observational study involving 6297 middle-aged and elderly individuals from a community population. Their investigation, which utilized the fatty liver index to define MASLD patients, revealed significantly elevated plasma ketone body levels in patients with MASLD compared to those without MASLD. Additionally, patients with higher ketone body levels exhibited an increased all-cause mortality rate [3]. This study consolidated previous research with much smaller sample sizes on the association between MASLD and ketone bodies in the general population, which had insufficient statistical power. For instance, Fotakis et al. conducted a study involving 210 individuals from both MASLD and non-MASLD groups in the general population. They found that the decrease in serum levels of acetoacetate and acetone was associated with MASLD risk in females, while no such association was observed in males [29]. Kotronen et al. compared the serum levels of β-hydroxybutyrate in 29 MASLD patients and 29 non-MASLD patients from the general population, but no statistically significant difference was found between the two groups [32]. In studies involving obese populations, Mey et al. found that MASLD patients among obese individuals exhibited significantly lower plasma levels of β-hydroxybutyrate compared to non-MASLD patients. They suggested that this observation might be related to skeletal muscle mitochondrial respiratory function [17]. Croci et al. revealed that MASLD patients who were overweight or obese showed a marked decrease in plasma β-hydroxybutyrate levels compared to lean non-MASLD individuals [16]. The research conducted by Robinson et al. further corroborated that, within obese populations, MASLD patients exhibited significantly lower serum levels of β-hydroxybutyrate and acetone compared to non-MASLD individuals [30]. Männistö et al. divided 116 obese patients into groups of normal liver, simple steatosis, and MASH, conducting a comparative analysis. They found that serum levels of acetoacetate and β-hydroxybutyrate were lower in MASH patients compared to those with simple steatosis. No significant difference was observed between steatosis patients and those with normal liver [31]. Based on previous large-scale research [3], it is inferred that MASLD patients exhibit elevated plasma ketone body levels compared to non-MASLD individuals. However, this relationship does not appear to hold true in obese populations and among patients with MASH, where blood ketone body levels do not increase but rather decrease. Further large-scale population studies for MASLD in obese populations and MASH are needed to confirm this observation. Additionally, a detailed investigation into the mechanism of the association between energy metabolism dysfunction and MASLD and MASH is warranted.

### 4.3. Ketone body levels with ALD

Long-term excessive alcohol consumption can lead to liver damage. Globally, approximately 3 million people die each year from ALD [44]. The liver damage associated with ALD includes three stages: alcohol-related fatty liver, hepatitis, and cirrhosis. Clinically, the incidence of alcohol-related ketoacidosis is significantly elevated among heavy drinkers, posing an urgent onset and high mortality rate.

As early as 1983, Stewart et al. conducted a comparative study involving 10 patients with alcohol-related cirrhosis, 5 patients with alcohol-related hepatitis, and 9 individuals. They found elevated blood glucose levels in heavy drinkers. However, due to the small sample size, the researchers did not observe differences in ketone body levels between heavy drinkers and the normal control group [35]. In 2014, Yokoyama et al. studied as many as 1588 alcoholic men in alcohol cessation centers and found that the prevalence of ketosis diagnosed by urinary ketone was as high as 34%. Furthermore, it was highly correlated with elevated liver enzymes and increased serum total bilirubin levels. The authors thus speculated that ALD might be associated with elevated blood ketone body levels [23]. Amathieu et al. and colleagues conducted a study of 93 stable alcohol-related cirrhosis patients in the intensive care unit and 30 liver failure patients. They confirmed that the serum ketone body levels in liver failure patients were significantly higher than those in the stable patients [33]. However, the study by Chen et al. found that the concentration of β-hydroxybutyrate in the liver is reduced in patients with alcoholic hepatitis [21]. On the other hand, clinically, the arterial ketone body ratio (AKBR, i.e., acetoacetate/β-hydroxybutyrate) can reflect hepatic reserve function and serve as a prognostic indicator for acute liver failure. Saibara et al. discovered that the AKBR may serve as a potential biomarker for ominous complications and adverse prognosis in patients with alcohol-related hepatitis [34]. Moreover, Cao et al. found that in patients with primary HCC, those with ALD had significantly higher tissue levels of acetoacetate compared to patients with primary HCC tumors associated with hepatitis B virus (HBV) or hepatitis C virus (HCV) infection. They also discovered that ketone-related metabolic pathways could serve as a basis for distinguishing the cirrhotic features of the primary HCC tumor. This approach may be able to determine whether it originates from ALD, HBV, or HCV infection [2]. Despite the prevailing belief that heavy alcohol consumption leads to liver damage and the notable increase in the incidence of alcohol-related ketoacidosis among heavy drinkers in clinical settings, blood ketone body levels are often used as clinical indicators of liver failure. There is currently no large-scale population study directly confirming the relationship between blood ketone body levels and chronic ALD. Further research is needed in this regard.

### 4.4. Ketone body levels with chronic viral hepatitis

Chronic viral hepatitis stands as one of the most pervasive liver ailments worldwide, primarily comprising hepatitis B and hepatitis C, triggered by HBV and HCV, respectively. As of 2019, 354 million persons are living with HBV or HCV infection globally. Of these, 87% are unaware of their infection, and 95% have not been treated [45]. HBV and HCV are transmitted through blood, bodily fluids, or sexual contact. Upon infection, they instigate inflammatory reactions, progressively compromising liver cell integrity and leading to impaired liver function.

Previous studies have suggested an association between ketone body levels and chronic HCV infection, yet the conclusions of related epidemiological research remain unclear. Sato et al. conducted a study comparing changes in serum insulin levels, HCV core protein levels, and blood ketone body levels after fasting in 30 patients with chronic hepatitis C and 8 healthy volunteers. They found a significant decrease in total blood ketone body levels in patients with chronic hepatitis C, along with impaired mitochondrial β-oxidation, possibly attributable to HCV infection [38]. Another study compared the serum metabolome in 67 HCV-infected individuals, 50 HBV-infected individuals, and 43 healthy controls. They found a significant increase in serum levels of β-hydroxybutyrate in HCV-infected individuals compared to both healthy controls and those with HBV infection [36]. Cao et al. conducted a study in 60 cirrhotic patients with primary HCC (20 with ALD, 20 with HBV infection, and 20 with HCV infection). They found that tissue levels of acetoacetate were significantly reduced in patients with primary HCC infected with HCV compared to those with HBV infection or ALD [2]. Embade et al.'s study involved 57 patients with chronic hepatitis C, including 30 non-fibrotic and 27 cirrhotic patients. The results revealed a significant downregulation of serum levels of acetoacetate and β-hydroxybutyrate in cirrhotic patients [37]. Current research has not yet identified a correlation between ketone body levels and HBV infection or other chronic viral hepatitis. This may be due to the lack of a pathological relationship between the two or the insufficient sample size and statistical power of existing studies to detect their correlation. Further epidemiological research is still needed to explore the relationship between ketone body levels and different chronic viral hepatitis types.

### 4.5. Ketone body levels with hepatic fibrosis

Hepatic fibrosis is a pathological self-repair response of the liver when it is subjected to long-term chronic injury. It is characterized by the abnormal deposition and distribution of extracellular matrix in the liver parenchyma. It represents a critical step in the progression of various CLDs to cirrhosis and is a significant determinant of prognosis in CLDs. Previous studies have indicated that MASLD patients are prone to developing liver fibrosis, which can progress to MASH and cirrhosis [10]. Furthermore, several large-scale population studies have confirmed that a greater degree of liver fibrosis is associated with a higher risk of mortality compared to the general population. This also suggests that the severity of liver fibrosis determines the severity of CLD [10],[46].

We did not find direct epidemiological studies on the correlation between the degree of liver fibrosis and ketone body levels. However, Lim et al. conducted a study involving 6202 individuals with MASLD. They found a negative correlation between ketonuria and the degree of liver fibrosis, which was independent of traditional metabolic factors [39]. Robinson et al. confirmed that in obese populations, the serum levels of β-hydroxybutyrate and acetone in patients with liver fibrosis were significantly lower than those in non-MASLD individuals. There was no difference compared to individuals with simple MASLD [30].

### 4.6. Ketone body levels with cirrhosis

Cirrhosis is an irreversible diffuse pathological change in the liver resulting from long-term damage. It represents the end-stage phase of various CLDs [47], with the most common causes including chronic viral hepatitis, ALD, and MASLD. When patients with cirrhosis experience decompensated liver function, they are at risk of life-threatening complications, making cirrhosis a common direct cause of death globally. Liver transplantation is the best and most definitive therapeutic option for patients with decompensated cirrhosis [48].

At present, epidemiological studies on the correlation between cirrhosis and ketone body levels are limited, with small sample sizes, and the conclusions are not yet clear. For example, in a study conducted by Liu et al. involving 42 patients with cirrhosis and 18 healthy controls, no significant differences were found in their serum ketone body levels [26]. The team led by Dabos et al. investigated plasma metabolomic data from 18 stable cirrhotic patients, 18 hepatic encephalopathy patients, and 17 healthy controls. They found elevated levels of acetoacetate and β-hydroxybutyrate in both stable cirrhotic patients and hepatic encephalopathy patients compared to the control group. Additionally, acetoacetate levels were higher in hepatic encephalopathy patients than in stable cirrhotic patients, while β-hydroxybutyrate levels were lower in hepatic encephalopathy patients than in stable cirrhotic patients [1]. Amathieu et al. found that in patients with severe ALD, the serum levels of acetoacetate and β-hydroxybutyrate were higher in patients with liver failure than in those with stable cirrhosis [33].

### 4.7. Ketone body levels with HCC

HCC, representing over 80% of liver cancer cases, contributes to liver cancer being among the top three causes of cancer death in 46 countries and among the top five causes of cancer death in 90 countries [49]. HCC presents a significantly elevated risk among patients with CLD. It often arises as a consequence of further progression of liver disease in patients with cirrhosis, but can also occur in patients with chronic viral hepatitis without cirrhosis [50].

A limited number of epidemiological studies have currently focused on the changes in ketone body levels in patients with HCC. Yan et al. conducted a study on 2143 patients with HCC and suggested that AKBR could serve as an indicator to reflect the postoperative metabolic status of patients [42]. Liu et al. studied 43 patients with HCC, 42 patients with cirrhosis, and 18 healthy controls. They found that compared to patients with cirrhosis and healthy controls, the serum levels of β-hydroxybutyrate were significantly elevated in patients with HCC [26]. Teilhet et al. found that in patients with HCC, the levels of β-hydroxybutyrate in the HCC tissue of patients with cirrhosis were higher than those in the HCC tissue of patients with MASLD. This suggests that mitochondrial dysfunction may be a mechanism for the progression of HCC [41]. Furthermore, Sasaki et al. studied 68 patients with HCC who underwent transcatheter arterial chemoembolization. They found that the levels of blood ketone bodies generally decreased after treatment in these patients. This was correlated with intramuscular fat content and may serve as a novel predictive marker for assessing the prognosis of patients undergoing surgery [40].

## 5. Hypothesis based on epidemiological synthesis

**Figure 3.**
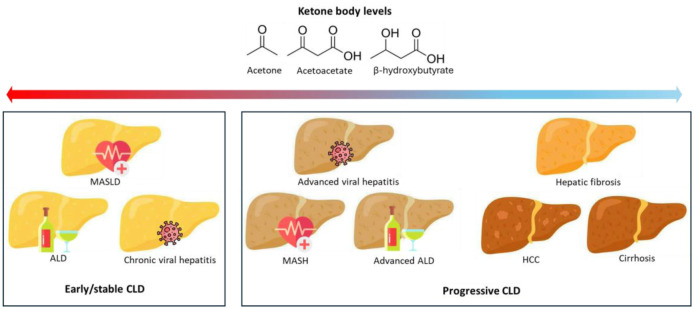
Ketogenesis hypothesis in CLD progression. Schematic of how ketone metabolism changes with advancing chronic liver disease. Red arrows/lines indicate a positive association between ketone body levels and disease risk; blue arrows/lines indicate a negative association.

The epidemiological evidence linking ketone body levels to various CLD reveals a biphasic and context-dependent relationship. We propose a unifying hypothesis: ketone body levels may serve as a biomarker reflecting the balance between hepatic metabolic adaptability and the severity of metabolic dysfunction. In stable disease, ketones may indicate active fatty acid clearance and potentially confer protective signaling. Conversely, in advanced disease, their dysregulation—either a decline from hepatic impairment or a pathological rise from systemic crisis—signals decompensation and worse outcomes (Figure 3).

### 5.1. The compensatory ketogenesis hypothesis in early/stable disease

This hypothesis posits that in the early stages of CLD (e.g., stable MASLD, chronic viral hepatitis), elevated ketone body levels primarily reflect a functional hepatic adaptive response. The liver enhances ketogenesis as a compensatory mechanism to manage increased fatty acid flux and prevent lipotoxicity [10]. The observed correlation between ketone body levels and MASLD likely originates from this robust compensatory function prior to significant liver injury. This theory may also explain inconsistent findings in studies on chronic viral hepatitis, as variations in hepatic compensatory capacity among patient groups could lead to different ketone body levels [36],[38]. The protective role of β-hydroxybutyrate in stable ALD, potentially mediated through receptors like Hcar2 on macrophages, further supports the idea that ketone bodies can be part of a beneficial adaptive signaling response in stable disease states [21].

### 5.2. The metabolic exhaustion and crisis hypothesis in progressive disease

As CLD progresses, this compensatory system fails, leading to a shift in ketone body dynamics. In the progression to MASH and advanced ALD, a critical metabolic shift might occur. With ongoing injury, insulin resistance and mitochondrial dysfunction impair the liver's ability to sustain compensatory ketogenesis. This “metabolic exhaustion” results in a decline from initially elevated ketone levels. Concurrently, disturbances in extrahepatic ketolysis may lead to metabolic disruptions that further promote hepatic inflammation, oxidative stress, and fibrosis, exacerbating liver damage [51],[52]. Similarly, in HCC, elevated ketone levels may not represent compensation but a hijacking of metabolism by tumor cells that utilize ketones as an alternative fuel to support rapid proliferation [53].

## 6. Therapeutic implications

The potential therapeutic relevance of ketones on CLD has received increasing attention, yet no clinical recommendations can currently be made regarding ketogenic interventions for CLD. The mechanisms involved are still not fully understood, and the intervention methods are also highly controversial.

In situations of hypoglycemia and glycogen depletion, ketones can serve as alternative substrates for hepatic metabolism, aiding in maintaining hepatic energy metabolism [54]. This process might theoretically be achieved by enhancing endogenous ketone body levels through hepatic ketogenesis to alleviate metabolic burden and inflammatory responses in patients with CLD. Exogenous intake of ketones can also supplement ketone body levels in the body. The main methods of endogenous ketogenesis include fasting, calorie restriction, ketogenic diet, exercise, etc. Exogenous sources of ketones include β-hydroxybutyrate supplements and natural foods rich in β-hydroxybutyrate, such as dairy products.

Weight loss is currently the most effective treatment for MASLD [55],[56]. Caloric restriction and exercise, as safe and effective weight loss methods, may help reverse the condition in MASLD patients. However, whether the mechanism of disease reversal depends on the action of ketones remains unclear. The other two endogenous ketogenesis methods, namely fasting and ketogenic diet, are still subject to much controversy regarding their safety as weight loss strategies [57]. Currently, there is no direct evidence or clinical support for the use of fasting, ketogenic diet, or exogenous ketone supplementation for the treatment or alleviation of CLD.

Many preclinical studies, especially animal studies, have shown that both endogenous and exogenous modulation of ketone body levels has protective effects on the liver, including anti-inflammatory, antioxidant, and hepatocyte regeneration properties [58]–[60]. However, these findings have not yet been validated in human trials, but they provide preliminary clues for future research on the therapeutic use of ketones in the treatment of CLDs. For example, numerous studies have shown that a ketogenic diet might protect the liver in mice, with mechanisms including alterations in hepatic mitochondrial flux, inhibition of the tricarboxylic acid cycle, and promotion of β-oxidation, among others [58],[59]. The potential application of a ketogenic diet in the treatment of HCC has also garnered attention [60]. The ketogenic diet may potentially inhibit the growth of HCC by regulating blood glucose and insulin levels, modulating lipid metabolism, and attenuating inflammatory responses in experimental models [60]. Additionally, Sarkar et al. found through mouse experiments that intermittent fasting can promote rapid proliferation of mouse hepatocytes [61]. Miyauchi et al. also observed in murine models that preoperative fasting for 12 hours can alleviate insulin resistance-induced liver inflammation and oxidative stress [62]. Bae et al. found that intraperitoneal injection of β-hydroxybutyrate in elderly rats can inhibit oxidative stress and formation of hepatic inflammasomes [63]. Chen et al. also observed the mechanism of alcohol-induced liver damage in mice by injecting β-hydroxybutyrate [21]. Further research is still needed to demonstrate whether food, as an exogenous source of ketone bodies, can alter the levels of ketone bodies in the body and induce liver-related pathophysiological changes.

## 7. Conclusions and perspectives

This review synthesizes evidence indicating a significant, yet complex, association between ketone body levels and common CLDs. Epidemiological studies generally suggest that ketone body levels are associated with MASLD and MASH, which may be explained by insulin resistance, BMI, diabetes, medications, and alcohol intake. The relationship between ketone body levels and other CLDs requires further confirmation through studies involving larger population samples. The mechanisms linking abnormal ketone metabolism to CLDs can be broadly categorized into two main types. First, dysregulation of ketone body levels may serve as a causative factor in CLDs by inducing hepatic inflammation, oxidative stress, and insulin resistance, leading to liver damage and the progression of CLDs. The second category involves the dysregulation of ketone body levels as a transient product during certain stages of the development of CLDs. For instance, reduced hepatic function leading to decreased ketogenesis may be reflected in blood ketone concentrations or the onset of stress responses in the body as CLDs progress to their terminal stages, resulting in a state of ketosis. Therefore, in further research on the mechanisms underlying the relationship between ketone body levels and CLDs, we need to focus on the causality between the two. This plays a crucial role in determining whether the regulation of ketone body levels could serve as a therapeutic target for CLDs in future studies.

We have to admit that the narrative nature of this review, while integrative, does not constitute a systematic review or meta-analysis. The associated limitation of Table 1 is that it is designed as a descriptive summary. We recognize it does not provide pooled quantitative estimates or a formal risk-of-bias assessment, reflecting the heterogeneity in study designs, populations, and analytical methods across the field. Its primary value is to offer a structured overview of the epidemiological landscape. However, the current evidence base remains observational and fragmented, highlighting a clear need for more standardized, prospective studies to establish causality and define clinical utility.

In summary, the relationship between ketone body levels and common CLDs is significant, but research in this area, whether epidemiological or mechanistic, remains insufficient. We believe that with the establishment of large-scale public databases such as the UK Biobank, we can better study the relationship between ketone body levels and various CLDs. The widespread adoption of causal inference methods such as Mendelian randomization can assist us in inferring the causal relationship between ketone body levels and various CLDs. The refinement of mouse models for various CLDs can help us better understand the mechanisms by which ketone body level regulation affects liver damage at the animal experimental level. The widespread adoption of drug precursor-targeted delivery carrier technology can further assist us in investigating the feasibility of clinical experiments on ketone body level regulation.

## Use of AI tools declaration

During the preparation of this work, the authors used ChatGPT solely for spelling/grammar checking and partial language translation assistance. The AI tool was not used to generate any research content, data analysis, or interpretative conclusions. The authors take full responsibility for the integrity of the work's intellectual content.
